# Energy current and computing

**DOI:** 10.1098/rsta.2017.0449

**Published:** 2018-10-29

**Authors:** Alex Yakovlev

**Affiliations:** School of Engineering, Newcastle University, Newcastle upon Tyne, UK

**Keywords:** electromagnetism, energy current, energy-modulated computing, Oliver Heaviside, Petri nets, transmission lines

## Abstract

In his seminal *Electrical papers*, Oliver Heaviside stated ‘We reverse this …' referring to the relationship between energy current and state changes in electrical networks. We explore implications of Heaviside's view upon the state changes in electronic circuits, effectively constituting computational processes. Our vision about energy-modulated computing that can be applicable for electronic systems with energy harvesting is introduced. Examples of analysis of computational circuits as loads on power sources are presented. We also draw inspiration from Heaviside's way of using and advancing mathematical methods from the needs of natural physical phenomena. A vivid example of Heavisidian approach to the use of mathematics is in employing series where they emerge out of the spatio-temporal view upon energy flows. Using *series* expressions, and types of natural discretization in space and time, we explain the processes of discharging a capacitive transmission line, first, through a constant resistor and, second, through a voltage controlled digital circuit. We show that event-based models, such as Petri nets with an explicit notion of causality inherent in them, can be instrumental in creating bridges between electromagnetics and computing.

This article is part of the theme issue ‘Celebrating 125 years of Oliver Heaviside's ‘Electromagnetic Theory’’.

## 1. Preface

This year we are celebrating the 125th anniversary of Heaviside's electromagnetic theory. For me personally, the turning point in terms of rediscovering Heaviside was the year 2013. Nearly 40 years had passed then since I started my Master's degree in Computer Engineering at what was the oldest Electrical Engineering school in the USSR, now called St Petersburg Electrotechnical University. The 5 year curriculum of the Computer Engineering degree included two substantive courses on Engineering Maths and on Electrical Circuit Theory. In those lectures, I had first heard of Heaviside in connection with the step function and operational calculus. For some reason, our curriculum did not contain a course on Electromagnetic Fields, though many of my friends doing other degrees such as Automatic Control or Electrical Measurements, let alone Radio Engineering, did Fields. Curiously, the national academic board of Computer Science degree studies felt that we, as future computer engineers, would not need to go as deep as the Fields. The Board probably thought that knowing Circuit Theory would be sufficient for all our practical needs, in order to understand how analogue and digital circuits work. Today, in hindsight, I realize that was an omission. I metaphorically talk about this ‘smart separation of concerns’ as building a ‘circuit theory wall', beyond which computer engineers should not go! Perhaps, indeed, most of the contemporary computer engineers and academics never need to look over this wall. Some knowledge of electromagnetics passes to them through a standard undergraduate Physics course, and everybody accepts that this should be enough for having a bigger picture of the electrical and electronic world.

Yet, in the year 2013, I came across Oliver Heaviside's work in full. How? From around 2010 I was involved in a large research project on the new generation of energy-harvesting electronics. The project embraced several research teams from different universities. The full set of expertise of these teams included power generation, power regulation and energy-harvesting-aware computational circuits. My team at Newcastle was responsible for the latter. It was during that project that I started to build the vision of computational electronics that was driven by potentially unpredictable and variable power sources rather than what had usually been seen as the recipient of unlimited power or energy supply. I called this type of computing ‘energy-modulated' [1], to emphasize the fact that it was energy flow that defined the computational action. In the following section on energy-modulated computing, I shall briefly explain its main idea. To underpin this concept, a patent on a self-powered voltage sensor was filed in 2011 [2]. In this method and apparatus, we used a capacitor to store the energy of the measured signal as a charge. This charge then was used to drive a self-timed frequency divider (or counter), which effectively converted the charge in the capacitor into binary code. At some point, the voltage across the capacitor became too low to drive the increment action in the counter. The resulting code could then be used as a digital measure of the initial voltage across the capacitor. An interesting challenge was to determine the strict relationship between this voltage and the code. Furthermore, another interesting question came up. What is the law according to which the charge, and hence the voltage across the capacitor, follows in time when the capacitor is discharged through a digital load formed by such a self-timed circuit? This answer was found and it appeared to be a hyperbolic (not exponential!) decay law—we presented in the following paper [3] in 2013. This derivation is reproduced in this paper.

Next, in the same 2013, by sheer coincidence I exchanged emails with Mr Ivor Catt about the late Professor David Kinniment, my colleague and mentor of many years, who studied an interesting and challenging phenomenon called metastability (connected to the philosophical problem of choice and the story of Buridan's ass) [4,5] in digital circuits during his 45-year academic career. From David Kinniment I had known that Ivor Catt was one of the early discoverers of this phenomenon, which he called The Glitch [6]. To my amazement, in my conversation with Ivor Catt, he told me about his other passion. That other work, which had absorbed him for nearly 40 years, was on developing and promoting his own version of electromagnetic theory (called Catt-theory or Theory C) [7]. Ivor Catt sent me his book and several articles in IEEE journals and in the *Wireless World* magazine. They showed how this theory advanced Heaviside's theory (Theory H) of transverse electromagnetic (TEM) waves and the concept of energy current. I managed to organize a seminar on Electromagnetism at Newcastle on 9 October 2013 to which I invited Mr Ivor Catt and Dr David Walton, who worked with Ivor Catt on various parts of his theory, particularly on demonstrating that a capacitor is a transmission line (TL) [8]. Coincidentally, David Walton obtained both of his Physics degrees from Newcastle University, and on the same day of 9 October 2013 there was a historical 50th anniversary reunion of Electrical Engineering graduates of 1963, some of whom had known David Walton (moreover some again, by coincidence had known Ivor Catt), so the date was truly momentous. Ivor Catt himself gave a 2 h lecture [9] which was followed by an hour-long lecture by David Walton [10]. These lectures showed a demonstration of the physics of some phenomena, ordinarily known to engineers, such as charging a capacitor, in an unconventional form—namely by applying a step voltage to a TL. The well-known exponential charging was the result of an approximated series of discrete steps caused by the cyclic process of the travelling TEM wave. This theory was supported by an experiment, known as Wakefield experiment [11], which led to the conclusion that there is no such a thing as a static electric field in a capacitor. In other words, a capacitor is a form of TL in which a TEM wave moves with a single fixed velocity, which is the speed of light in the medium. Below we reproduce both the derivation of the TL-based capacitor discharge and the description of the Wakefield experiment.

Those lectures triggered my deep interest in studying Oliver Heaviside's work and, even more, his whole life. And this very interest drew me to (then PhD student but now Dr) Christopher Donaghy-Spargo, with whom we founded NEMIG—northeast Electromagnetics Interest Group, which since 2013 has enjoyed a formidable series of seminars given by scientists, engineers, historians and entrepreneurs, driven by the ideas and lives of Maxwell, Heaviside and generally by the exciting field of electromagnetism.

Coming back to the main object of this paper, which is the relationship between energy current and computing, I must admit that I had drawn most of inspiration from my familiarization with Heaviside's work, his legacy in the work of others, and to a great extent by the fact that both Ivor Catt and David Walton came to studying electromagnetic theory from the point of view of energy current through their experiences in dealing with high-speed digital electronics. This electronics does not deal with sine waves. It deals with digital pulses, which are physical enough to be dealt with in a ‘more physical way' rather than expressing them as an algebraic sum of sine wave harmonics stretching in the time domain from −∞ to +∞. Such pulses have a clear starting point in time and endpoint in time. They naturally lend themselves to causality between actions, such as a rising edge of one pulse causes a falling edge of another pulse, for example, as the signal passes through a logic NOT element (inverter). As I spent most of my own 40 working years exploring asynchronous self-timed digital circuits, and such circuits could work directly when the power is applied to their vdd lines, I was firmly attracted by the natural beauty of the ideas of the electromagnetic theory approach relying basically only on energy current, Poynting vector (*S* = *E* × *H*, vector product of the electrical field vector and magnetic field vector, representing the directional energy flux, measured in Watt per square metre; note that it is sometimes referred to as Umov–Poynting vector) and TEM wave—particularly by its compactness and parsimony of Occam's Razor.

Another important aspect of my fascination of the energy-current approach to computational electronics is associated with the fundamental role that mathematical series play there. Series, so much loved and revered (to the poetic level!) by Heaviside, are at the core of the vision of all electromagnetic phenomena because they relate all state changes in the electromagnetic field with the geometry of the space and medium. Likewise, series form the foundation to the definition of main measurements of the dynamic fields and hence for the subsequent discretization and quantization important for the computational procedures. This paper pays particular attention to the use of mathematical series, their constructions, summations and other analytical operations. By doing so, this paper would like to pay homage to Heaviside as a magician of the series mathematics.

Setting the scene, I would like to finish this preface with a quote from David Walton's lecture abstract [10]:

It is normally recognised that the postulation of Displacement current by James Clerk Maxwell was a vital step which led to the understanding that light was an electromagnetic wave. I will examine the origins of displacement current by consideration of the behaviour of the dielectric in a lumped capacitor and will show that it has no physical reality. In the absence of an ether there is no rationale for displacement current. We are then left with a theory which works mathematically but has no basis in physical reality. I will discuss the remarkable property of empty space in that it has the ability to accommodate energy. I will then show that Faraday's law and conservation of charge lead to the existence of electromagnetic energy which travels at a single fixed velocity and has a determined relationship between the electric and magnetic fields. Because this mathematics is reversible it follows that these two physical laws can be considered to be consequences of the nature of electromagnetic energy rather than the reverse.

## 2. Energy-modulated computing

The question we pose here is: *How does energy drive computations?*

The traditional way of making computers perform calculations is to connect a processor, memory and all the interfaces to the outside world to a power supply. The power supply is a source of energy, which must always be THERE and ON and deliver as much power (energy per time unit) as the computer needs to run its programs and interact with the environment. Typically, these needs are determined by the rate of activity of the processor, or more specifically by the frequency at which the clock generator runs the processor. In traditional systems, the ‘motive force' for the processor comes from the clock generator under whose tick-events the hardware of the computer updates its state. This way of building computer systems considers power supply and timing mechanisms, both needed to the processor, separately, and such a separation requires significant effort (including power and time costs) to make sure that the power level always matches the frequency, otherwise the computer will produce erroneous results. With this approach, which has been dominating computer engineering for many years, any considerations of energy usage are usually in context with energy consumption, which is seen as an effect of the performed computational actions rather than cause thereof.

Now, instead of separating power and timing sources, we can think of the processor's own timing mechanism that can be powered by the same power supply as the rest of the system. It will then turn the whole system into an energy-driven machine, which will be driven by the energy flow, pretty much like any biological organism functions in nature. Traditionally, the relationships such as Power = *f*(*F*, *V*), where *F* is the switching frequency and *V* is the supply voltage, have been studied and the figure of merit has been, for example, Watt/MegaHertz. In energy-driven approach we are more interested in the relations such as Delay = *f*(*V*) or Delay = *f*(*P*), or Rate = *f*(*V*), hence the figure of merit is MegaHertz/Watt or MegaHertz/Volt.

**Figure 1.**
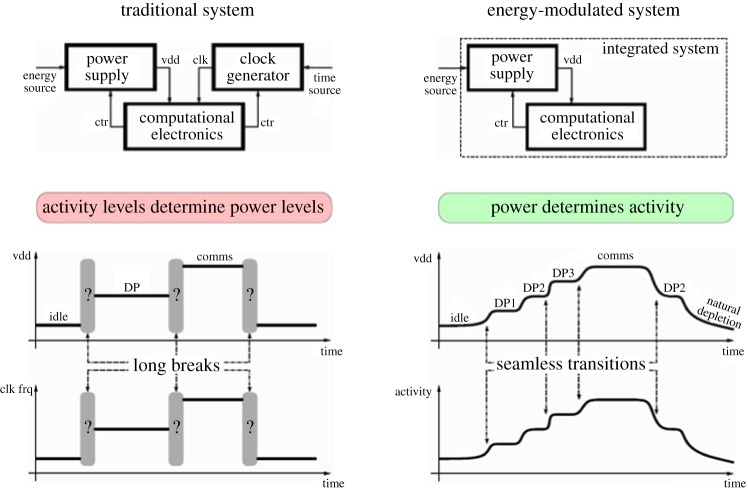
Traditional versus energy-modulated view upon system design. (Online version in colour.)

Figure 1 illustrates the above distinction, by showing two designs of a functionally same system, which switches between different modes, idle, data processing and communication. Clearly, the traditional way is significantly less robust to possible variations of supply voltage vdd, which either makes the system error-prone or inefficient with lots of interruptions. This view upon the computing systems being an exemplar of physical systems, rather than purely mathematical objects, i.e. products of human imagination, brings us to a most inspiring quote from Heaviside's work [12]:

Now, in Maxwell's theory .. the potential energy … and … the kinetic or magnetic energy are supposed to be set up by the current in the wire. We reverse this; the current in the wire is set up by the energy transmitted through the medium around it. The sum of the electric and magnetic energies is the energy of the electric machinery which is transmitting energy from the battery to the wire. It is definite in amount, and the rate of transmission of energy (total) is also definite in amount.

Although this reversal of the viewpoint mostly concerned the analytical aspect of Electrical Science, it can be used today as a system design principle or paradigm. Real-power computing [13] describes hardware and software systems subject to a ‘real-power constraint', for example, the availability of energy from a power source, or restriction on power dissipation. Real-power applications must guarantee performance within specified definite power constraints, referred to as ‘power bands’. Systems of this type are linked to the notion of survivability, which depends on their power aspects as well as their ability to morph and retain functional aspects to ensure continued computation. Real-power systems go well beyond conventional low-power systems that are optimized for minimum power consumption only.

The energy-modulated approach to system design permits building systems in which information and energy flows to the system are not structured as fundamentally different thereby, again, bringing our computing engines closer to natural conditions. Traditionally, they are supplied separately, which makes them scale poorly with modern technology. Namely, either there is much energy unused where there is no need for processing information, or information is lost when energy is not available. Contrary to this, we can actually supply information with energy in bundles, hence leading to *computing with energy*.

**Figure 2.**
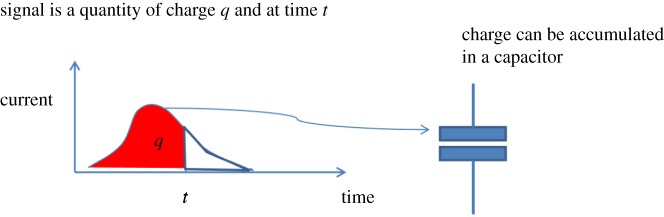
Catching the energy of a signal in a capacitor. (Online version in colour.)

In this spirit, we can structure information and energy in the form of tokens (cf. Heavisidian pulse-based view). An information token must consist of Data that is Powered (Energy Validity) and Ready (Timing Validity). Here we can associate such a token with a signal in the form of an electric current which can be integrated as a charge in a capacitor (figure 2). In our subsequent sections, we will show how this charge can be turned into a computable object or datum, i.e. for example measured and digitized. It should be noted that a signal which contains timing and power validity is both self-timed and self-powered (STSP). A practical way to process such signals in electronics is to use asynchronous circuits [14], where researchers have built a significant body of knowledge since the late 1950s.

**Figure 3.**
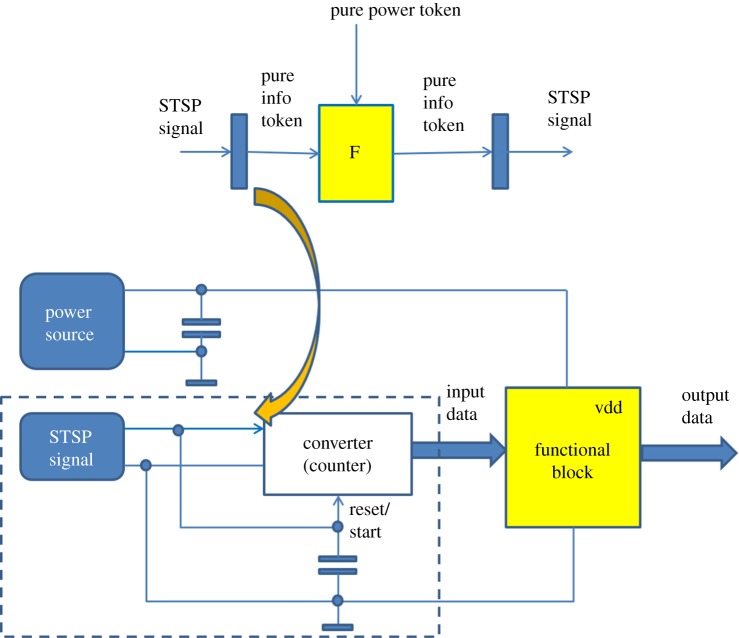
Processing self-timed self-powered signals. (Online version in colour.)

This idea is schematically shown in figure 3. As we show further in this paper, computing can be involved at the level of energy current or at the level of the accumulated energy, such as electric charge. What one can see here is an STSP signal, or several such signals, that trigger processing in the functional block. The triggering action can be tuned to very small scale energy levels, and instead of using traditional amplification, which requires energy-costly separate power regulator, one can use integration of charge above a certain threshold. (Fundamentally, integration is also far more noise-immune than amplification!) With modern technology scaling, this approach can help us avoid two main shortcomings of the conventional computing, namely (i) unused energy when there is no need for processing, and (ii) lost information when energy is not available.

What's crucial for energy-modulated computing is the notion of causality, discussed below.

### (a) Energy current and causality

Interestingly, the view in which energy flow is seen as a cause to performing action goes back to Galileo and Newton, who emphasized the role of geometry in representing causality and dynamics in nature. Dr Ed Dellian, who translated and edited the key works of Galileo and Newton, wrote to me (E Dellian 2018, personal communication):

I have learned that the matter requires to distinguish carefully between arithmetic (algebra) and (Euclidean) geometry. Galileo's saying in the ‘Saggiatore' (1623) refers to geometry as the ‘art of measuring' what there ‘really is’. Arithmetic (algebra) on the other hand is ‘the art of calculating' what there ‘should be'. In order to understand the importance of the art of measuring in natural philosophy, that is, geometry as the ‘language of nature', one must consider what Newton says in his foreword of 1686 to the ‘Principia'. This is at least as relevant as Galileo's dictum in the ‘Saggiatore’: For Newton, geometry is the general basis of all of ‘mechanics'. He calls geometry ‘that part of mechanics in general which demonstrates and teaches the art of exact measuring’.

To us, pursuing the above energy-modulated viewpoint, measuring and computing are synonymous. It is therefore important to see the distinction between geometric (physical) and algebraic (metaphysical) views on nature, with the former building on and expressing causality and causal proportionality while the latter being non-causal, and what is sometimes called tautological proportionality. To this end, Dellian further wrote:

You know already that this ‘E', the symbol of ‘cause', is not equivalent to the classical energy ‘E'. Rather it is the same thing as Galileo's and Newton's ‘vis impressa', the cause of (change of) motion (cf. Newton's def. 4). Note that Newton explicitly says to have taken his first and second law from Galileo!

In other words, we have two ‘kinds’ of energy—one is *creative and causal*, and the other is purely *characterizational*. In the following, a series of statements summarize my understanding of Dellian's analysis and its relation to electromagnetic theory:

1. Energy current (**E-vector**) causes **momentum *p***.
2. **Causality** is made via the proportionality coefficient *c* (**speed of energy current, i.e. the speed of light**)
3. Momentum *p* is what mediates between E-vector and changes in the **matter**.
4. Momentum *p* is preserved as energy current hits the matter.
5. Momentum in the matter presents another form of energy (**E-scalar**).
6. E-scalar characterizes the elements of the matter as they move with **a (material) velocity**.
7. As elements of the matter move, they cause changes in Energy current (E-vector) and this forms a *fundamental feedback mechanism* (which is recursive/fractal …).

Telling this in terms of **electromagnetic theory and electricity**:

1. E-vector (Poynting vector/Heaviside signal) causes E-scalar (electric current in the matter).
2. This causality between E-vector and E-scalar is mediated by momentum *p* causing the motion of charges.
3. The motion of charges with material velocity causes changes in E-vector, i.e. the feedback effect mentioned above (e.g. self-induction).

We can now conclude that the key aspects for energy-modulated computing, inspired by Heaviside's notion of energy current, are the following:

— Capturing energy current that moves in space with speed of light into material form (possibly electric charge in a capacitor) to enable measurement and hence information processing.
— Detecting the completion of such process in the form of validity information.
— Discretizing information in time in the form of steps and cause–effect relationships between signals.

Thus computation can take place at different levels, at both energy current level as well as at the material level. As we show in the following sections the important role in defining computation at both these levels mathematically is in the use of series. Such series characterize the process of energy accumulation and division in space and in time.

## 3. Computing by accumulating and dividing energy

### (a) On the creative role of *series*

Use of functional series was essential for Heaviside's mathematical toolbox. He wrote in [15]:

The subject of the decomposition of an arbitrary function into the sum of functions of special types has many fascinations. No student of mathematical physics, if he possesses any soul at all, can fail to recognise the poetry that pervades this branch of mathematics.

This is an inspiring statement and the energy current approach to the analysis of electronic systems is exactly what encourages one to use series. Why? This is because the energy current carrying any change in the electromagnetic field always moves with a speed of light (which is a constant for a given medium), and all processes of changing the state of the field can be seen in the form of spatial division, or discretization, so that exact time intervals between them can be determined from the spatial values and the speed of light. It is then very handy to use simply the indexation of steps in iterative processes associated with the changes of the states. One of the best illustrations of this methodology comes with the analysis of TLs. In the following section, we will illustrate this with the use of the energy current approach in describing the process of discharging a capacitor through a constant resistance. The capacitor will be modelled by a lossless TL. I find this analysis, which is reproduced from Catt *et al.* [16], highly illuminating. In this analysis we avoid modelling a TL as a set of distributed RC parameters, and only rely on the notion of a TEM wave travelling in the medium between two metal wires (or, equivalently, plates of a capacitor) with a speed of light, as postulated by Catt's electromagnetic theory [7].

### (b) Capacitor as transmission line

**Figure 4:**
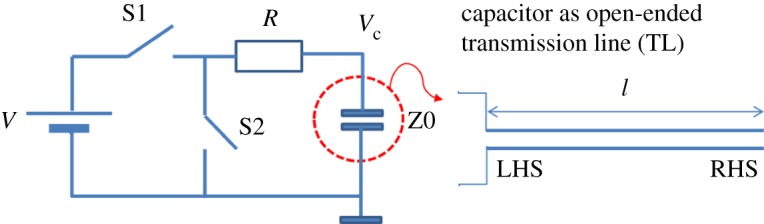
Circuit for charging and discharging a capacitor seen as a transmission line. (Online version in colour.)

The configuration that we want to consider here is shown in figure 4.

Assume, first, that the capacitor was charged via resistor *R* to the voltage *V* (via switch S1). Then we disconnect S1 and connect S2. The capacitor is a (e.g. coaxial cable) TL with a characteristic impedance *Z*0. Let us assume that *R* ≫ *Z*0, and we assume that *R* is constant. The reflection coefficient at the right-hand side terminals of the open-ended TL: *ρ* = (*R* − *Z*0)/(*R* + *Z*0). So, at time *t* = 0, we have S2 in the ON state, while S1 is in OFF state, and 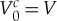. At this moment a down-step of magnitude (*Z*0/(*R *+ *Z*0))*V* starts to propagate down the TL. As it is open on the right-hand side it is reflected with a coefficient 1 and is seen on the left-hand side as 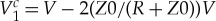. After *n* two-way passes of the TEM wave:
3.1
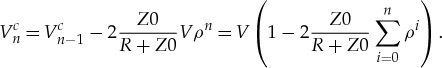


Denote *a* = 2 (*Z*0/(*R *+ *Z*0)) and, because *ρ* < 1 we can use the sum of geometric progression for 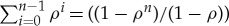. Therefore, we have 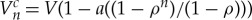. However, *a*/(1 − *ρ*) = 1, hence we have
3.2
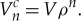


It is now clear that the discharge process is exponentially decaying. In the long run, 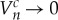 as *n* → ∞.

Now we can show that this exponential process can be expressed in the form *e*^−(*t*/*τ*)^, which is customary to standard lumped capacitor discharge analysis. Consider the term
3.3
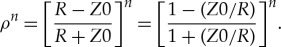


If, as we assumed, *Z*0/*R* ≪ 1, then *ρ^n^ *≈ [1–2(*Z*0/*R*)]*^n^*. Define *k* = 2(*Z*0/*R*)*n*, and hence *ρ^n^* ≈ [1 − (*k*/*n*)]*^n^*. So,
3.4
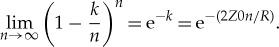


Now let us connect the index of step *n* with time *t*. At time *t*, *n *= *vt*/2*l*, where *v* is the velocity of propagation, i.e. the speed of light in the medium, and *l* is the length of TL. Hence, 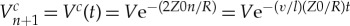. Now, for any TL, we know that 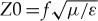 and 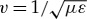, where *μ* is the permeability and *ϵ* is the permittivity of the dielectric medium between the plates of the TL and *f* is the geometric factor. This geometric factor relates a linear unit capacitance *c* with *ε*, as follows: *ε* = *cf*. So, for the TL of length *l*, the total capacitance is *C* = *cl*. Substituting all these elements into (*v*/*l*)(*Z*0/*R*) = 1/RC, and finally, we have *V^c^*(*t*) = e^−(*t*/RC)^. In other words, thanks to the two important series that we summed, a geometric series and the power series representing a natural exponential, we have been able to derive the approximation of the step-wise process driven by energy current captured in the space defined by the TL. This process is equivalent to the process of discharging a lumped capacitor of capacitance *C* through a lumped resistor of resistance *R*.

Interestingly, the appropriateness of modelling capacitors with fast switching as TLs has been demonstrated experimentally by Sullivan & Kern [17]. Without resorting to fundamental arguments, such as Heaviside's energy current or Catt theory, they observed the following:

The ideal transmission line model exaggerates the definition of the steps in the voltage waveform, particularly after more than one round-trip time. If one simply evaluates rms error between the simulated waveform and the actual waveform, one might think that the ideal transmission line is a poor model, and that more accurate transmission line models, as begun in section V-D, are necessary before transmission line models can be useful to the designer. We intend to work on such improved models, but we believe that the simple ideal transmission line model will often be more useful to the designer. It provides a conceptual model of what behavioural features to expect, and relates those features directly to geometry. In particular, the height and timing of the first and second steps are probably the most important features. The height of the first step is given by *Z*_0_Δ*I* for the height of the first step, and twice that for the second step.

### (c) The Wakefield experiment

**Figure 5.**
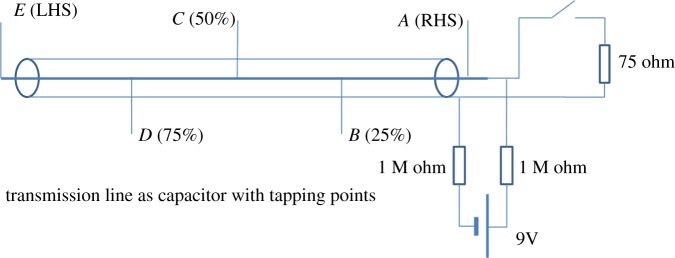
Wakefield experiment set-up: coaxial cable as a cap with tapping points. (Online version in colour.)

An experimental evidence of the stepwise discharge process for a capacitor modelled by a co-axial cable has been presented by Ivor Catt in *Electronics World* in April 2013 [11]. Here is only a brief recap of this description. The experiment bears the name of Mr Tony Wakefield of Melbourne, who actually built the configuration and performed all the measurements. Catt wrote:

We now have experimental proof that the so-called steady charged capacitor is not steady at all. Half the energy in a charged capacitor is always travelling from right to left at the speed of light, and the other half from left to right [see figure 5].

The Wakefield experiment uses a 75-ohm coax 18 meters long. The left-hand end is an open circuit. The right-hand end is connected to a small, 1 cm long, normally open reed switch. On the far side of the reed switch is a 75-ohm termination resistor simulating an infinitely long coaxial cable. A handheld magnet is used to operate the switch.

The coax is charged from a 9 V battery via 2 × 1 megohm resistors, close-coupled at the switch to centre and ground. The two resistors are used to isolate the relatively long battery wires from the coax. High value resistors are used to minimize any trickle charge after the switch is closed.

A 2-channel HP 54510B digital sampling scope set to 2 V div^−1^ vertical and 20 ns div^−1^ horizontal is used to capture five images.

**Figure 6.**
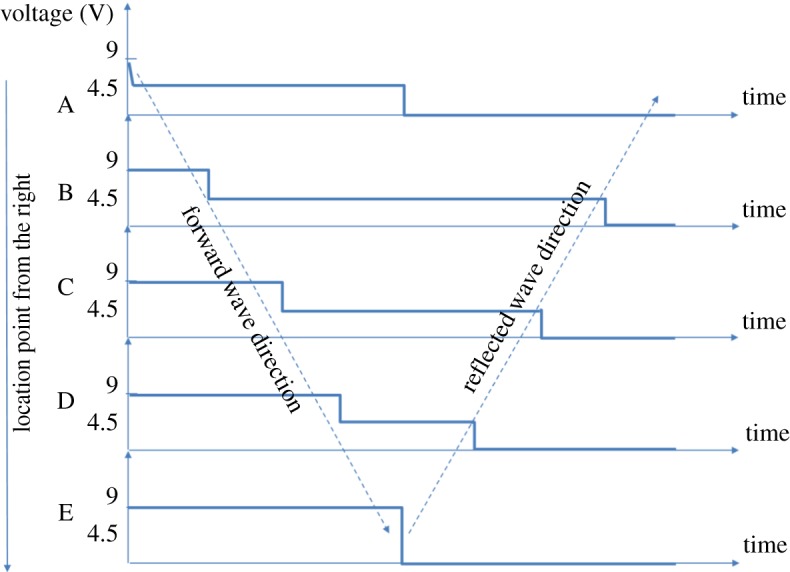
Signal plots for the Wakefield experiment, in five different locations. (Online version in colour.)

For the reasons of copyright, I cannot copy these images from Catt's paper. But, they were taken in the following points: (A) across the terminator 75-ohm resistor, (B) 25% to the left of the reed switch (4.5 m), (C) 50% to the left of the reed switch (9 m), (D) 75% to the left of the reed switch (13.5 m), (E) at the extreme left of the open end of the cable (figure 6).

### (d) What if the load is a self-timed digital circuit?

When I asked myself a question of what the law of discharging a capacitor via a self-timed switching circuit is, I had already suspected that it was not a normal exponential process that we see in an RC circuit with a constant *R*. Clearly the fact that the switching circuit forms a voltage-controlled impedance would affect the discharging process. Theoretically, one can try to produce a model of resistor *R* and apply it at every step of the discharging process. But then the question arises, what if the dynamics of change of *R* is not related to the dynamics of the capacitor discharge? While it would still be interesting to study the discharging process for a TL in place of a lumped *C*, here we can consider the model of discharging a lumped capacitor via a digital switching circuit as the first step in this direction. Here, we will focus on the step-wise process associated with the changes in the resistive load due to switching from one destination capacitor to another capacitor and modelling the dynamics of the delay in such switching. As a self-timed digital switching circuit, we consider a ring oscillator. The original details of this derivation can be found in papers with my colleagues Dr Reza Ramezani, Dr Alex Kushnerov and Dr Andrey Mokhov [14,18].

**Figure 7.**
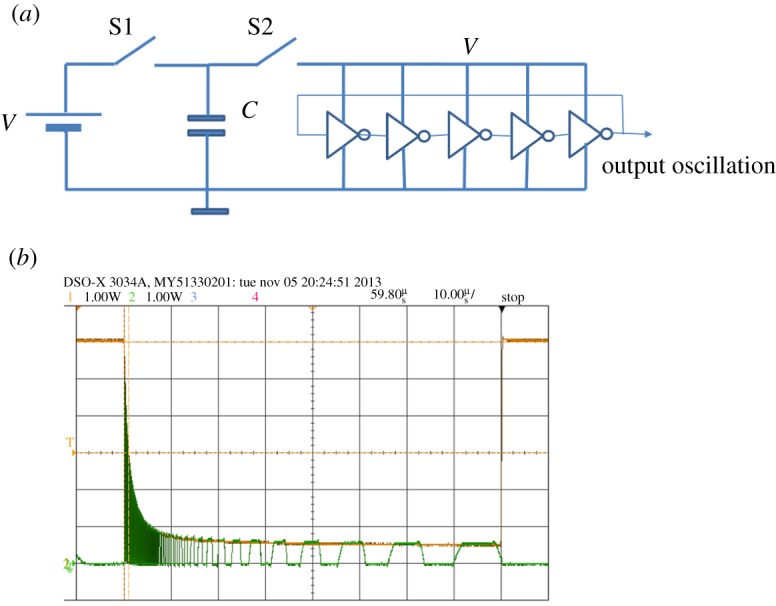
Ring oscillator powered by a capacitor (*a*) and its behaviour inverters (*b*), showing both the voltage drop and the output oscillation. (Online version in colour.)

**Figure 8.**
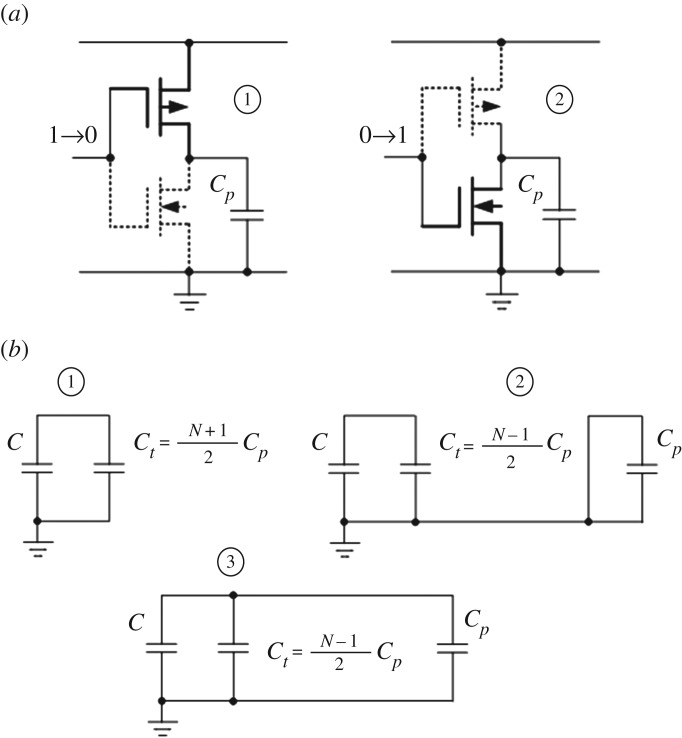
Charge switching: transistor switching (*a*), and the circuit state at dynamic switching (*b*), where *N* is the number of inverters.

Let us consider a capacitor and a ring oscillator built out of an odd number of inverters, as shown in figure 7*a*. With two switches we can first charge the capacitor from a voltage source (switch S1 On and Switch S2 Off), and then discharge it by powering up the ring oscillator (switch S1 Off and Switch S2 On). A switching process in any digital circuit, built from CMOS gates, is a process of charging and discharging its parasitic capacitances through PMOS and NMOS transistors, respectively. For a ring oscillator, this behaviour proceeds in series, between each pair of adjacent inverters, wherein the preceding inverter, after charging its parasitic capacitance from the power line, activates the input of the next inverter, which pulls down its output, thereby discharging its parasitic capacitor, and so on. This process is illustrated in more detail in figure 8*a*, where it is shown that the pull up (charging) is done via the PMOS transistor in the ON state and the pull-down (discharging) via the NMOS transistor in the ON state. The charging of each such parasitic capacitance *C_p_* is by taking part of the charge from the main capacitance *C*.

Because there is always only one inverter active, we can describe the operation of the ring oscillator and the main capacitor in three states, as shown in figure 8*b*. Configuration 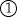 represents the circuit state when no charging or discharging action takes place. Half of the inverters (say, odd-numbered) are at logical ‘1', i.e. their corresponding capacitors *C_p_* are charged to the voltage level equal to that of *C*. The remaining inverters are at logical ‘0' and their capacitors *C_p_* are empty. This state also describes the circuit status between two successive switching events. The system is in state 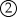 when a switching occurs. One of the charged capacitors *C_p_* (of the inverter which is supposed to switch from 1 to 0) is discharged via the corresponding NMOS transistor. In state 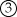 the discharged capacitor associated with the next inverter in the ring (which is supposed to switch from 0 to 1) receives charge from the source capacitor. This is the only state which draws energy from the source capacitor. Note that, in real operation an overlap exists between states 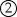 and 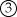, however, to simplify the analysis we consider them as distinct states.

At the level of abstraction where we consider configurations shown in figure 8, we would normally deal with the exponentials involved at each state of charging and discharging the current parasitic capacitor. As discussed in the previous section if we look at the main capacitor as a TL, such exponentials are actually step-wise processes. In this section we shall abstract away from those TL-related processes, assuming that their dynamics is much faster than the delays associated with the states shown in figure 8. Instead, we will go one level of abstraction higher and look at the steps associated with the transitions between such states. Thus we will concentrate only on the charge division at each step, which we call the *V* drop. The timing aspect, i.e. the propagation delay involved in the charging and discharging state, can be considered separately. The *V* drop in this circuit can be found by the law of conservation of charge, which redistributes between the capacitors *C_T_* = *C* + *C_t_* and *C_p_* according to their capacitances. The voltage across the main capacitor can be written as a recurrence relation that is unfolded then into an iterative process with the initial condition of *V*_0_.

**Figure 9.**
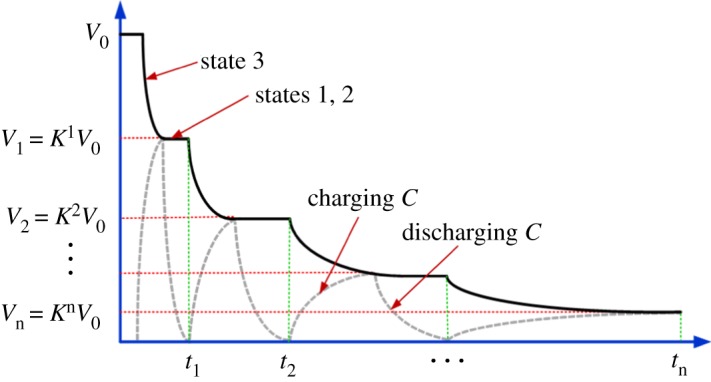
The *V* drop over time, with charging and discharging of parasitic capacitors in the inverters. (Online version in colour.)

Thus, at the *n*-th step, charge equilibrium occurs at
3.5
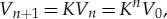

where
3.6
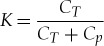

is the coefficient of voltage decay at each switching action (or ‘charge division coefficient'). Since *C_T_* ≫ *C_p_*, *K* is only slightly less than one. Thus, the switching index *n* determines the voltage at each step. This process is shown in figure 9, where for the sake of better illustrating the effect of charge division we exaggerate the ratio so that *K* is much smaller than one. Let us now consider the delay of such steps. The propagation delay of an inverter is a function of supply voltage, which determines the behaviour of its NMOS and PMOS transistors. For operation above the transistor threshold (super-threshold region), the time of switching (propagation delay) is approximately reciprocal to *V*.

However, below threshold (sub-threshold region), the propagation delay becomes a more complex function of *V*. The following is a model for the propagation delay of a single inverter proposed in [19]:
3.7
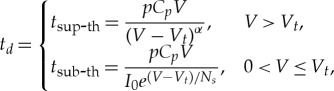

where *p* is the fitting parameter, *α* is the velocity saturation index (which we consider here for simplicity to be equal to 2), *I*_0_ is the drain current at *V_gs_* = *V_t_* and *N_s_* = *mkT*/*q* (*m* is the sub-threshold slope factor, 1/*N_s_* ≈ 28) [20]. In the following, we will consider, for simplicity, the operation in the super-threshold region and approximate the propagation delay as *t_d_* = *A*/*V*, where *A* = *pC_p_*. The overall elapsed time at step *i* is conveniently represented by the sum of the individual propagation delays of the steps. Thus the physical time *t* is also determined by the increments of the switching index:
3.8
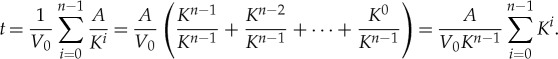


This adds up all the time intervals *t_d_* that precede *n* and represents the total time spent on the first *n* switching events. We know that *K < 1*, so we have finally the sum of a geometric progression:
3.9
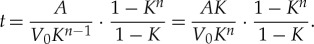


And if we consider that after the *n*-th switching event *V_n_* = *V*_0_*K^n^* we can substitute it into (3.9) and obtain
3.10
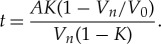


Now, solving this equation with respect to *V_n_*, we obtain it as a function of time
3.11
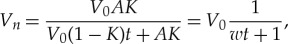

where
3.12
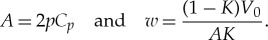


This characteristic is a hyperbola rather than exponential as it would have been if the capacitor was discharged through a constant resistance. Note again that the hyperbola of the kind of (3.11) is the result of considering the behaviour of transistors in the inverters of the circuit in the super-threshold region. In near-threshold and sub-threshold regions, the character of the discharging process is fairly complicated due to the exponential increase of the delay as voltage drops, and this significantly reduces the rate of the voltage drop due to logic switching. Eventually, the switching process stops. The remaining charge decay is mainly determined by the leakage current.

In this analysis, performed in a Heaviside way, an intermediate factor, called a switching index *n*, was introduced to simplify the process of deriving the important relationship between the voltage on the capacitor *V* and time. Notably, both time and voltage of the power supply of the circuit are thus quantized according to the switching index *n*, rather than using a fixed time discretization step as often happens in numerical analyses of circuits. Voltage is dropping exponentially with *n*, and time is stretched as a function of the supply voltage, or in other words, of the energy available in the main capacitor. In consequence, the overall time is obtained by accumulating the intervals of switching in the form of generating functions. Solving these functions provides analytical solutions which express voltage as a function of (global) time. Using the discrete parameter *n* as an intermediate step appears to be a useful ‘trick' in deriving the relationship between the two characteristics, voltage and time, that are themselves continuous.

On the basis of the idea of discharging a capacitor via a self-timed circuit, we were able to build charge-to-digital converters (CDC) which could be used to measure capacitance or voltage. For this, we simply had to replace the ring oscillator with a counter of switching activity [21]. In those paper, we showed that, either we could use the self-timed counter directly as an oscillator-counter, or have a delay chain and a separate counter.

**Figure 10.**
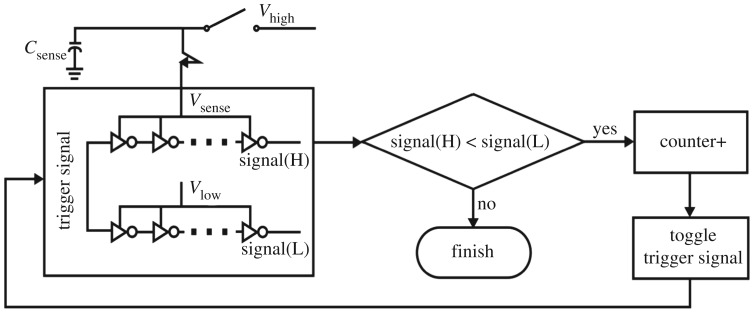
The idea of building a sensor using a charge to digital converter.

**Figure 11.**
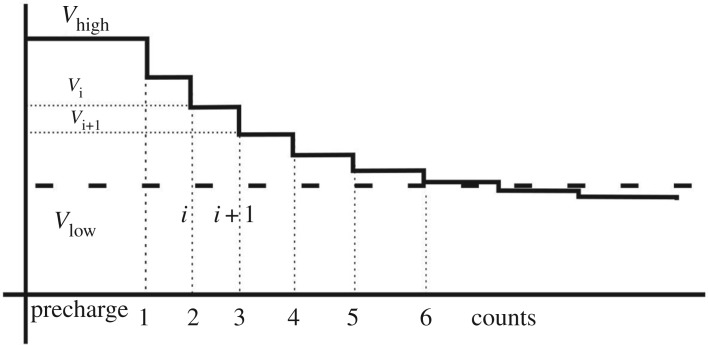
Discharging and counting process.

Figures 10 and 11 show how to build a CDC using an iterative delay chain discharge mechanism. An unknown capacitance *C* is charged to an initial voltage level *V_H_* and then discharged to a pre-defined reference voltage level *V_L_*. The number of iterations of discharging is then accumulated in a counter and it is related to the initial voltage level *V_H_* and the value of *C*.

Let *k* = (1 − *K*) = *C_p_*/(*C_T_*+*C_p_*) ≈ *C_p_*/*C_T_*, because *C_T_* ≫ *C_p_*, where *C_T_* = *C* + *C_t_* and *K* was defined by equation (3.6). The step-wise capacitor discharge process was described above and led to the equation:
3.13
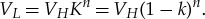


Based on Taylor series, (1 − *k*)*^n^* = 1 − *nk* + *n*(*n* − 1)*k*^2^/2! − *n*(*n* − 1)(*n* − 2)*k*^3^/3! + ⋯ and, if *nk* ≪ 1, the above formula can be approximated as (1 − *k*)*^n^* = 1 − *nk*. From the fact that we have levels *V_H_* and *V_L_* fixed (i.e. *const*) by the measurement method, we must have (1 − *k*)*^n^* = 1 − *nk* = *const*. Thus, under *nk* ≪ 1, *nk* = *const*, and hence
3.14
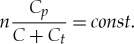


So, if *C* ≫ *C_t_*, then *C* + *C_t_* ≈ *C*. Thus, *C_p_*/*C* = *const*, which means *n* must be linearly proportional to *C*. The value of *n,* if accumulated in the counter, can be used to obtain the value of a measured capacitance *C*.

Using similar construction, we can also measure voltage *V_H_* if we know the capacitance value *C*. Suppose we also know *V_L_*. Therefore, since *V_L_* = *V_H_K^n^*, after the number of discharging steps *n*, the latter can be determined as
3.15
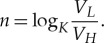


Thus, *n* is logarithmic with the measured voltage *V_H_*.

### (e) On quantization and discretization: hypotheses

In this section, I will consider some rather interesting, and possibly controversial, implications of the transients that we visited above thanks to Catt, Davidson and Walton's derivations. The artefact that those transients had envelopes that were exponential or sine/cosine curves was the result of having them been sums of series of steps in the first place. Furthermore, they originated as series of steps from one, rather simple but fundamental, postulate—that of the existence of energy current that is never stationary but always moves with the speed of light (Catt's Theory of [7]).

Understanding this postulate and the various analyses of transients in electrical systems is important. It is crucial for settling with the idea of the world being quantized by virtue of energy currents being trapped between some reflection points, and the continuous pictures of the transients are just the results of some step-wise processes.

I deliberately use word ‘quantized' in the above because I tend to think that ‘quantization’ and ‘discretization' are practically (in the physical sense; mathematicians may argue of course because they may add some abstract notion to these terms) synonyms. So I will try to explain my point below.

Let us see what happens with the TEM as it works in a TL with reflection. We have a series of steps in voltage which eventually form an exponential envelope (with a linear time-invariant resistor). If we examine these steps, they show discrete sections in time and amplitude. The values of time sections between these steps are determined by the finite and specific characteristics of the geometry of the TL and the properties of the (dielectric) medium. The value of the amplitude levels between these steps is determined by the electrical properties of the line and the power level of the source. So, basically, these discrete values associated with the energy entrapment in the TL are determined by the inherent characteristics of the matter and the energetic stimulus. If we stimulated the TL with periodic changes in the energy current, we could observe the periodic process with discretized values in those steps—the envelope of which could be a sequence of charging and discharging exponentials. Likewise, if we set up a TL (which is largely capacitive in the above) with an inductance, so we will have an LC oscillator; this would produce a periodic, similarly step-wise, discretized process whose envelope will be a sine wave (see [7]).

Now, if we analyse such a system in its discretized (rather than enveloped) form, we could produce some sort of histogram showing the distribution of how much time the object in which we trap energy current, spends in what level of amplitude (we could even assign specific energy levels). Now, we can call such an object a ‘Quantum Object'. Why not? I guess the only difference between our ‘quantum object' and ones that Quantum Physicists are talking about would be purely mathematical. We know the object well and our characterization of the discretized process is deterministic while in Quantum Physics the exact evolution of an object between discretized states may be unknown, hence probabilities are employed (interesting polemic issues may arise here as I seem to touch the territory of the EPR paradox [22]).

On the basis of these arguments, I would like to make some hypotheses.

We live in the world that has finite size objects of matter, whatever large or small they are. These objects have boundaries. The boundaries act as reflection points on the way of the energy current. Hence associated with these objects and boundaries we have entrapments of energy. These entrapments, due to reflections give rise to discretization in time and level. The grains of our (discretized) matter can be quite small so the entrapments can be very small and we cannot easily measure these steps in their sequences, but rather characterize by some integrative measurements (accumulate and average them—like in luminescence), hence at some point we end up being histogrammatic or probabilistic.

One more thing that may still bother us is the verticality of steps and their slopes.

Let us look at the moment when we change the state of a reed-switch or pull up the line to vdd or down to Ground. The time with which this transition takes place is also non-zero. I.e. even if the propagation of the change is with the speed of light, modulo the ‘epsilon' and ‘mu' of the medium, i.e. with finite time to destination, the transition of the voltage level must also be associated with some propagation of the field, or forces, inside the reed-switch or in the transistor, respectively, that pulls the line up or down. Clearly that time-frame is much smaller than the time frame of propagating the energy current in the medium along the TL, but still, it is not zero. I presume that, recursively, we can look at the finer granularity of this state change and see that it is itself a step-wise process of some reflections of the energy current in that small object, the switch, and what we see as a continuous slope is actually an envelope of the step-wise process.

So ultimately, we live in the recursive or fractal world of quantized space and the current limit of what we can measure as a step is something defined by, say, electron tunnelling microscope.

These ideas are pretty much in line with the state of the art knowledge in chemical physics and modern positions of some leading physicists on ‘classical' Quantum Mechanics [23]. For example, from my discussions with Prof Werner Hofer of Newcastle University, I came to the understanding that electron is a portion of space, surrounding the nucleus of an atom, which has trapped energy current, pretty much analogous to a capacitor!

## 4. Mathematical models for energy-modulated computing

### (a) Modelling Wakefield experiment in Petri-nets

In this section, I would like to make a brief excurse to a computational model called Petri nets. The name that this model bears is attributed to Prof. Carl Adam Petri, whose work [24] on causal models of information processing and communication using a graphical formalism of condition-event nets in the 50s and early 60s had led to a significant amount of research and applications in computer science and beyond. Petri nets have a very clear distinction between locality and nonlocality. In fact, in Petri nets, one can express cause–effect relationship between discrete events in a very succinct form, even if one has many events occurring concurrently and independently in space. I spent nearly 40 years studying different types of Petri nets and their semantics and applying Petri nets to the analysis and design of electronic systems.

What is a Petri net? It is a bipartite directed graph [25] with nodes being either places (denoted by circles) or transitions (denoted by bars). Sometimes places are also called conditions and transitions are called events. The arcs of the graph, called a flow relation, can only go from places to transitions and transitions to places. These arcs define the causality relation between transitions, i.e. events, via places. Places, transitions and arcs define the structure of the net. A Petri net can produce a dynamic behaviour, which is defined by the initial position of token in some places (called initial marking or initial state) and some rules of a ‘token game', i.e. the method according to which the current marking can change into the following marking and thus produce sequences of markings. The token game has two main rules. Rule 1 (Enabling Rule) defines the condition under which a transition is enabled. Namely, a transition is enabled if all its input places (places that are connected to the transition by arcs going from the places to the transition) contain tokens. Every transition that is enabled under a given marking can fire. Rule 2 (Firing Rule) says that when a transition fires, we have to remove a token from each input place and add a token to each output place (such places are connected by arcs going from the transition to places). On the basis of these rules, one can perform exploration of the reachable markings (called reachability analysis). This exploration, done systematically and exhaustively, requires performing algorithmic search techniques, such as depth-first search or breadth-first search. The size of the reachable state space may grow exponentially if the Petri net has transitions that are enabled *concurrently* in the same marking, because all possible interleavings of firing such transitions need to be explored. The important advantage of Petri nets themselves as a model for representing distributed concurrent systems is their compactness. On the contrary, the reachable state space represented by the so-called reachability graph would have all concurrency captured in the form of interleaving sequences, and hence suffers from complexity burst.

**Figure 12.**
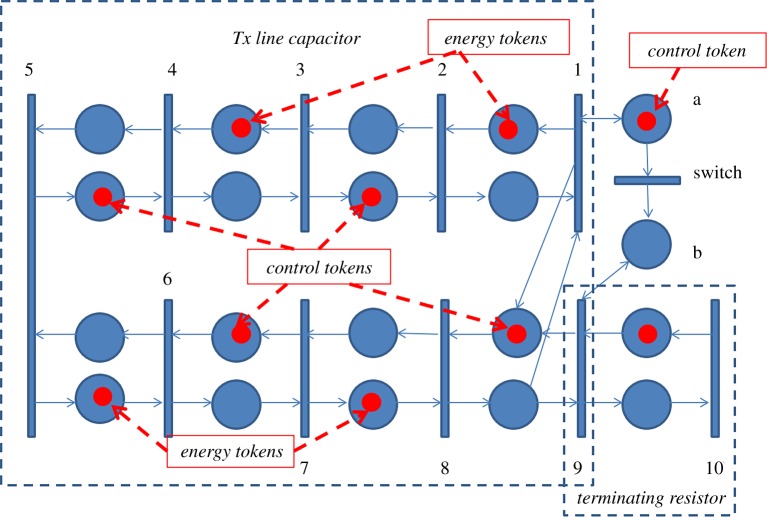
Petri net model of a transmission line with a switch and terminating resistor (Wakefield experiment).

We shall avoid discussing formal definitions of the Petri net structure and behaviour (there are several semantics of concurrency and choice that can be associated with this behaviour). Instead, we shall simply proceed to the use of Petri nets for the modelling of the distributed system associated with the TL and energy current moving in it taking place in the Wakefield experiment [11]. The system previously shown in figure 5 is now modelled by the Petri net in figure 12. This net captures the energy current by tokens in the upper and lower threads—they are called ‘energy tokens’. These tokens move in the upper subnet right-to-left and in the lower subnet left-to-right to demonstrate the effect of the direct (from the switch to the far end) and reflected TEM waves (the wave that moves to the right will again be reflected back to form another direct wave after some loss of energy into the subnet modelling the terminating resistor). Specific transitions can be associated with signal events in particular locations of the cable. Namely, transitions 1 and 9 stand for events in point A, 2 and 8 in B, 3 and 7 in C, 4 and 6 in D and 5 in E. The Petri net of such kind is usually called a ‘ring pipeline net'. Ring pipeline conveniently shows the cyclic rotation of the energy tokens in the cable medium.

**Figure 13.**
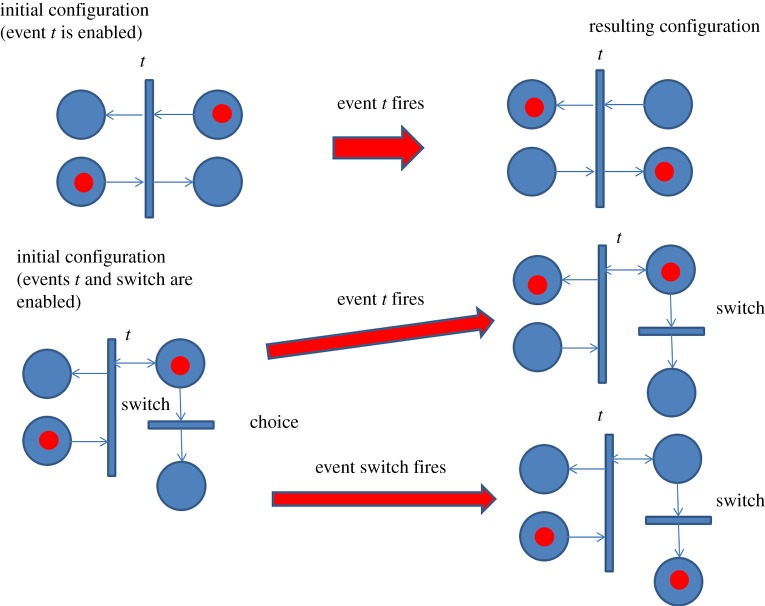
Illustration of Firing Rule for transitions in the Petri net of figure 12.

Note that this net can operate cyclically without any outgoing tokens to the resistor while the ‘control token' initially sits in the place labelled ‘a'. This place enables transition 1, which helps to sustain cyclicity in the process. This cyclic behaviour does not change the potential of the cable as it stays charged at 9 V. As soon as the switch is operated, the ‘control token' moves from place to ‘a' to place ‘b', and this activates the transition 9. When the control token is in place ‘b' transition 1 cannot be enabled anymore because there is no more token in place ‘a'. The firing rules for the main transition types used in this net are illustrated in figure 13.

**Figure 14.**
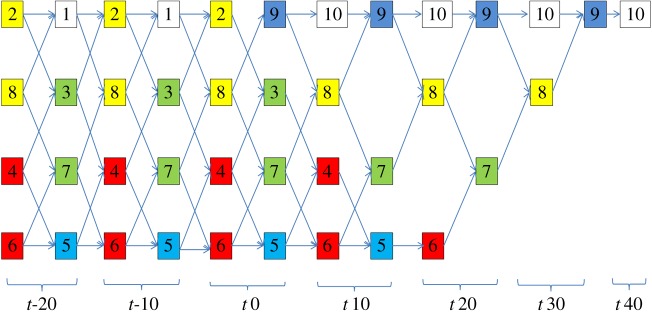
Petri net behaviour unfolding.

The behaviour of the Petri net model and its interpretation in terms of the states of the signal in the five main points of the cable can be analysed using an acyclic unfolding of the Petri net, whose fragment is shown in figure 14.

**Figure 15.**
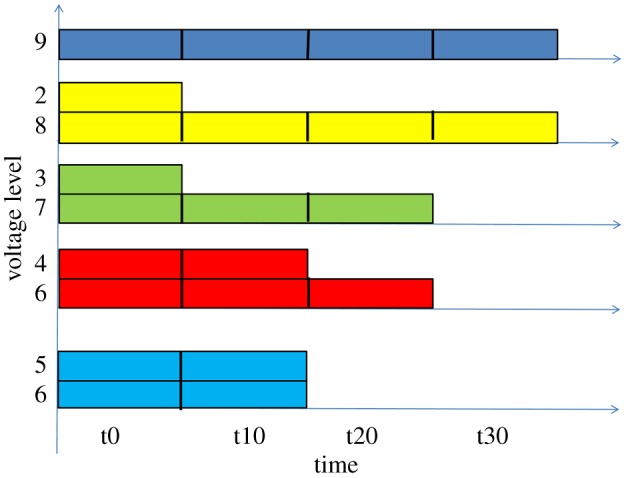
Waveform interpretation of the Petri net behaviour (cf. figure 6).

The initial segment of the unfolding is labelled with time stamps *t*-20 and *t*-10, which correspond to the time before the switch is ON. This shows the repetitive operation of pairs of transitions associated with points A (1), B (2 and 8), C (3 and 7), D (4 and 6) and E(5). Not all of these transitions are concurrent (this is because of the way we represented the pipeline—we alternate transitions passing the energy and control tokens—in principle we could show a denser pipeline but this would have required using some dummy events), but all of them fire in two adjacent batches at t-20 and t-10, which models the passage of the two cycles of rotation of energy current in the cable. At time stamp t0, we now have the first occurrence of event 9 (no longer 1) to represent action in point A. We can now trace the section of the unfolding which is marked by the trace of events: 2, 3, 4, 5 (direct wave), 6, 7, 8, 9 (reflected wave) taking place in the steps t0, t10, t20 and t30. The end of the signal propagation is at time t40, where transition 10 fires indicating that the initial energy level has left the cable through the terminating resistor. The Petri net unfolding can be interpreted as a waveform (with the colours of the corresponding transitions in the net), shown in figure 15. There is a distinct similarity with the waveforms from the scope in the Wakefield experiment shown in figure 6. Some discrepancy is caused by a bit coarse level of granularity with which we modelled the pipeline of the signals in the cable. Increasing granularity of modelling, i.e. discretizing the space and using more transitions would have improved the matching effect. But this would have cluttered the diagrams, so we avoided that here.

Petri nets are generally a good model to model repetitive group behaviour which was explored throughout many years by Carl Adam Petri. In the recent paper by Prof. Rudiger Valk [26], Petri's cycloids are introduced with a concept of slowness. As the paper claims, this approach can be applied to Organization, to Work Flow (Just-in-time Production), and to Physical Systems. It would be interesting to investigate how Petri nets and cycloids can capture such behaviour for example as propagations of slivers of pulses in groups of TLs.

## 5. Conclusion

More than 125 years ago Oliver Heaviside stated that energy current was the primal standpoint. In this paper, we looked at the potential impact of the idea of energy current on the connection between electromagnetic theory and computing. This connection is manifold. It permeates through the notion of energy-modulated computing. It also drives the research into computing which is based on physical phenomena such as causality and encourages the engineers to develop or use the ‘right kind' of mathematics to build the bridge between the behaviour of signals in physics and exploiting this behaviour in computations. The bridge between the physics of electromagnetism and computing fundamentally lies in Time domain analysis and appropriate forms of discretization of processes in space and time (cf. geometric approach of Galileo and Newton [27]). Immediate switching to Frequency domain analysis for pulse-based signals (and this is what we deal with in computers!) would bring a ‘wrong type' of mathematics on the way of physics and reality. This sounds controversial but this is what we could and should learn from Heaviside.

What about more specific methodological innovation of this paper? We have now explored two types of step-wise physical processes that we can link with computing. One is associated with energy-current—this is a fast computing paradigm associated with the speed of light. An example is the energy-division in TLs—here we can form oscillations at super-Gigahertz frequencies on a chip. Another form is associated with the switching of logic gates, where we rely on mass effects such as movement of charge, and division of electrical energy associated with it. This is illustrated by the capacitor discharge via digital switching logic. Here our typical speeds are sub-Gigahertz. These two forms are orthogonal but can work together, for example in a nested way, like the second and minute hands of the clock. We could combine the TL discharge (step-wise discretization of an exponential—inner loop) with a logic circuit switching (step-wise discretization of hyperbolic discharge—outer loop).

This is a conjecture with which I conclude this paper. It is based on the stepwise process of TL models of capacitors by Ivor Catt and his associates and our stepwise processes with a ring oscillator discharging a capacitor, even a lumped one. These are two orthogonal discretization operators. The study of their superposition is a subject of our future work. This will open up some new dimensions for energy-modulated computing!

Besides, a potentially useful result of this paper in terms of modelling is the fact that Petri net unfolding can be interpreted as a waveform of signals whose states are associated with some places in the net.

## Data accessibility

This article has no additional data.
