# Correction to ‘Energy current and computing’

**DOI:** 10.1098/rsta.2018.0351

**Published:** 2019-01-07

**Authors:** Alex Yakovlev

*Phil. Trans. R. Soc. A* **376**, 20170449. (Published online 29 October 2018). (doi:10.1098/rsta.2017.0449)

In figure 6, there is an error with one of the waveforms. The correct version of figure 6 is below.

**Figure 6.**
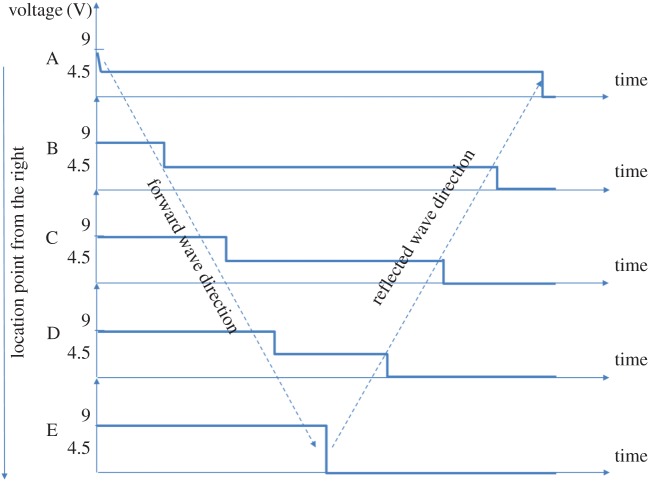
Signal plots for the Wakefield experiment, in five different locations.

